# Reduced P53 levels ameliorate neuromuscular junction loss without affecting motor neuron pathology in a mouse model of spinal muscular atrophy

**DOI:** 10.1038/s41419-019-1727-6

**Published:** 2019-07-04

**Authors:** Natalie L. Courtney, Alannah J. Mole, Alison K. Thomson, Lyndsay M. Murray

**Affiliations:** 1 Centre for Discovery Brain Sciences, Edinburgh Medical School: Biomedical Sciences, Edinburgh, UK; 2 Euan MacDonald Centre for Motor Neurone Disease Research, University of Edinburgh, Edinburgh, Scotland EH8 9XD UK

**Keywords:** Cell death in the nervous system, Diseases of the nervous system

## Abstract

Spinal Muscular Atrophy (SMA) is a childhood motor neuron disease caused by mutations or deletions within the *SMN1* gene. At endstages of disease there is profound loss of motor neurons, loss of axons within ventral roots and defects at the neuromuscular junctions (NMJ), as evidenced by pathological features such as pre-synaptic loss and swelling and post-synaptic shrinkage. Although these motor unit defects have been widely described, the time course and interdependancy of these aspects of motor unit degeneration are unclear. Recent reports have also revealed an early upregulation of transcripts associated with the P53 signalling pathway. The relationship between the upregulation of these transcripts and pathology within the motor unit is also unclear. In this study, we exploit the prolonged disease timecourse and defined pre-symptomatic period in the *Smn*^*2B/*^^−^ mouse model to perform a temporal analysis of the different elements of motor unit pathology. We demonstrate that NMJ loss occurs prior to cell body loss, and coincides with the onset of symptoms. The onset of NMJ pathology also coincides with an increase in P53-related transcripts at the cell body. Finally, using a tamoxifen inducible P53 knockout, we demonstrate that post-natal reduction in P53 levels can reduce NMJ loss, but does not affect other aspects of NMJ pathology, motor neuron loss or the phenotype of the *Smn*^*2B/−*^ mouse model. Together this work provides a detailed temporal description of pathology within motor units of an SMA mouse model, and demonstrates that NMJ loss is a P53-dependant process. This work supports the role for P53 as an effector of synaptic and axonal degeneration in a die-back neuropathy.

## Introduction

Spinal Muscular Atrophy (SMA) is a childhood form of motor neuron disease that is caused by homozygous loss of the *SMN1* gene^1,2^. The most common form of this disease has an onset of less than 6 months of age and a life expectancy of under 2 years without significant respiratory support.

The classic morphological features of motor unit pathology in SMA have been extensively characterised and widely used to assess outcome in pre-clinical testing^3^. They include a loss and cytoplasmic shrinkage of motor neuron cell bodies, loss of axons in the ventral roots and pathology at the neuromuscular junctions (NMJ), including denervation, pre-synaptic accumulation of neurofilaments, endplate abnormalities, and muscle fibre atrophy^4,5^. Such defects have been consistently observed in both patients and animal models of SMA at symptomatic stages of disease. How these pathologies throughout the motor unit are temporally related to each other, and to the onset of symptoms, is currently less clear.

It is thought that motor neurons degenerate in a ‘die-back’ manner, as the pre-synaptic terminal appears to withdraw from the post-synaptic endplate, leaving partially occupied endplates^6^. Furthermore, ventral root axon loss appears to be more severe than motor neuron cell body loss, which is indicative of distal to proximal degeneration of the motor neuron^7^. NMJ defects occur prior to symptom onset in mouse models of SMA suggesting that the NMJ is an ‘early pathological target’ in SMA^6,8^. However, the temporal and causative relationship of NMJ degeneration to the loss of motor neuron cell bodies is unknown.

It may be suggested that the observed defects at the NMJ in SMA mosue models are reflective of a requirement for Smn in the axon or synapse. However it is currently unclear whether synaptic pathology is a cause or effect of motor neuron death, and it remains possible that synaptic and axonal pathology observed are morphlogical evidence of cell death, as the motor neuron retracts its most distal processes. This idea is supported by the osbervation that there is an increase in transcripts associated with the P53 signalling pathway in motor neuron cell bodies at pre-symptomatic time points in SMA mouse models^8^. As this upregulation is more prominent in selectively vulnerable motor neurons, P53 may play a role in the breakdown of the motor unit^8,9^.

P53 plays a crucial role in co-ordinating the cellular response to stress. Initially characterised as a transcription factor, more recent work indicates it can also act as a translational regulator and interact with regulatory RNAs^10–12^. P53 is activated in response to a variety of cues including cell stress and DNA damage, and directs cellular responses, including senescence, DNA repair and apoptosis^13^. Although best characterised as a tumour suppressor, P53 has also been strongly implicated in a range of neurodegenerative disorders, including the adult onset motor neuron disease amyotrophic lateral sclerosis (ALS)^14–19^. An increase in P53 has been reported in postmortem patient spinal cord^20^ and an increase in transcripts associated with the P53 signalling pathway have been widely reported in motor neurons and spinal cord tissue from SMA model mice^8,21–24^.

The cause and consequence of P53 upregulation in SMA motor neurons is currently unclear. Given its well-established involvement in pro-apoptotic pathways, it is tempting to speculate that this upregulation is reflective of the onset of cell death pathways at the motor neuron. However, a direct interaction between Smn and P53 has also been observed, which was suggestive of an anti-apoptotic role for Smn^25^. The increase in the P53 signalling pathway may therefore be reflective of a primary defect caused by reduced Smn levels. However, since P53 activity appears restricted to selectively vulnerable motor neurons, it is also possible that P53 acts downstream of Smn, in mediating the breakdown of selectively vulnerable motor neuron pools. P53 has also been shown to localise to the synapse where it plays a role in mitochondrial dysfunction and oxidative stress following DNA damage^26^. P53 has also been shown to regulate a range of synaptic genes and that synaptic activity can regulate P53 levels^27,28^. It is therefore unclear what, if any, role P53 has in the onset and progression of motor unit pathology in SMA.

Here, we use the *Smn*^*2B/*^^−^ mouse model of SMA to demonstrate that the onset of NMJ degeneration precedes motor neuron cell body loss, thus confirming that motor neurons degenerate in a distal to proximal manner. We demonstrate that the first evidence of pre-synaptic swelling at the NMJ coincides with an increase in P53 associated transcripts in the spinal cord. In order to investigate the causal effects of P53 pathway activation, we have crossed the *Smn*^*2B/*^^−^ mouse model with a mouse carrying a tamoxifen inducible P53 knockout. Postnatal knockout of P53 led to a reduction in NMJ loss, with no benefit observed upon any other features of NMJ pathology, motor neuron loss or mouse phenotype. In summary, we show that NMJ loss is a consequence of P53 pathway activation, and that partially preserving the structure of the NMJ confers no benefit to the overall health of the motor neuron or mouse. P53 therefore appears to play a role in the breakdown and degeneration of synaptic connections, but does not appear to be central to the motor unit pathology caused by depleted Smn levels.

## Results

### NMJ pathology precedes motor neuron cell body loss in the *Smn*^*2B/*^^−^ mouse model

To investigate the correlation between NMJ and cell body pathology, we have performed a detailed temporal analysis of the NMJ and motor neuron cell body throughout disease in the *Smn*^*2B/*^^−^ mouse model of SMA^29^. The aim of this work was to give insight into the causal relationship between pathology in these distinct parts of the motor neuron.

In our hands, the *Smn*^*2B/*^^−^ mouse model displays a significant reduction in body weight from around P10, and motor deficits (as evidenced by an increase in the time taken to self right) which are evident at around P9 (Supplementary Fig. 1). It has a life expectancy of around 20 days.

**Fig. 1:**
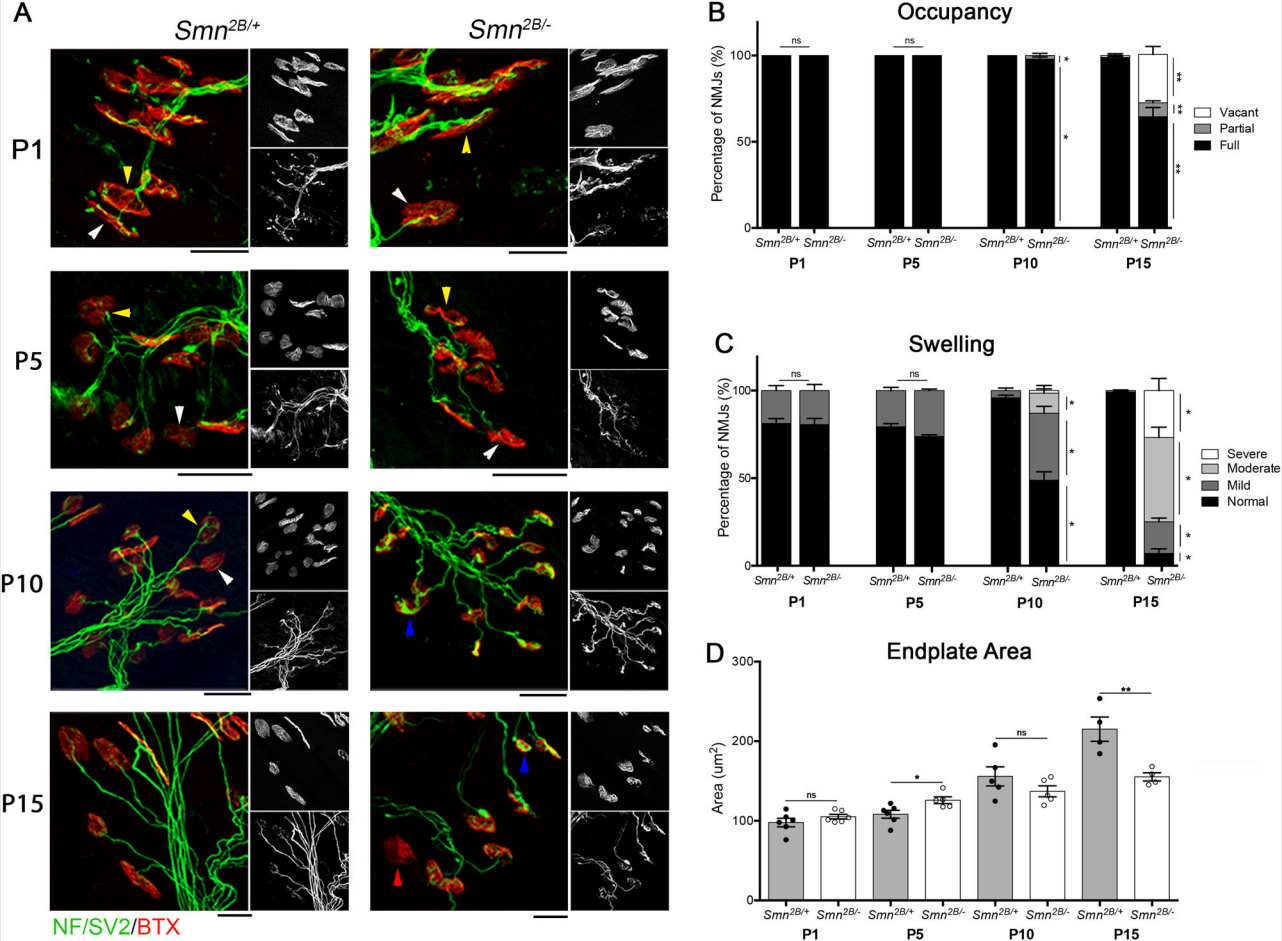
The onset of NMJ pathology is between P5 and P10 in the *Smn*^*2B/−*^ mouse model of SMA. **a** Representative confocal images showing maximum intensity projection of confocal z-stack of NMJs in the TVA of *Smn*^*2B/−*^ and *Smn*^*2B/+*^ mice at P1, P5, P10 and P15. Note that white arrowheads show no pre-synaptic swelling, yellow arrowheads show mild pre-synaptic swelling while blue arrowheads show moderate pre-synaptic swelling. Vacant endplates (red arrowhead) are seen at P15 in the *Smn*^*2B/−*^ mouse model of SMA. The grayscale images show, from top to bottom, BTX and NF/SV2. Scale bar = 20 μm. **b** Bar chart (mean ± SEM) compares the percentage of full, partial and vacant endplates in *Smn*^*2B/−*^ mice compared to *Smn*^*2B/+*^ controls. At P1 and P5, 100% of endplates in *Smn*^*2B/−*^ and *Smn*^*2B/+*^ mice were fully occupied by the pre-synaptic terminal. At P10, there is significant increase in the percentage of partially occupied endplates and at P15, there is a significant increase in the percentage of both vacant and partially occupied endplates in *Smn*^*2B/−*^ compared to *Smn*^*2B/+*^ mice (by Mann–Whitney-U test, *p < 0.05, **p < 0.01; *n* = 4 mice per genotype). **c** Bar chart (mean ± SEM) compares the stages of pre-synaptic swelling in *Smn*^*2B/−*^ and *Smn*^*2B/+*^ mice. There is no significant difference in the level of swelling in *Smn*^*2B/−*^ mice compared to controls at P1 and P5. At P10, there is a significant increase in the percentage of NMJs with stage 2 and stage 3 swelling in *Smn*^*2B/−*^ mice. At P15, there is a significant increase in the percentage of pre-synaptic terminals with stage 2, 3 and 4 swelling in *Smn*^*2B/−*^ mice compared to *Smn*^*2B/+*^ controls (by Mann–Whitney-U test, *p < 0.05; *n* = 4 mice per genotype). **d** The graph (mean ± SEM) compares the average size of endplates in the TVA of *Smn*^*2B/−*^ mice compared to controls. At P1 and P10 there is no significant difference in the area of post synaptic endplates in *Smn*^*2B/−*^ mice compared to *Smn*^*2B/+*^ controls. At P5, there is a small but significant increase in endplate area in *Smn*^*2B/−*^ mice. At P15 there is a significant decrease in the size of endplates in *Smn*^*2B/−*^ mice compared to controls (unpaired t-test, *p < 0.05, **p < 0.01; *n* = 4 mice per genotype)

NMJ pathology was assessed in the transversus abdominis muscle (TVA), a muscle which has demonstrated significant defects across all mouse models of SMA which have been studied to date^6,29,30^. Consistent with previous reports^29^, analysis of the TVA muscle at P15 revealed significant pre-synaptic pathology, as evidenced by a decrease in the percentage of fully occupied endplates and significant increase the percentage of NMJs displaying mild, moderate or severe swelling (Fig. 1a–c). A timecourse analysis of these markers of pre-synaptic pathology revealed that the earliest time point at which they could be detected was at P10. This was the first time point at which denervation could be detected, as evidenced by a small but significant increase in the percentage of partially occupied endplates. There was also a significant increase in the percentage of NMJs with mild or moderate pre-synaptic swelling. There was a trend towards a decrease in endplate size at P10, which was statistically significant by P15 (Fig. 1a–d). Overall, this data suggests that the onset of NMJ pathology occurs after P5 but is present by P10.

As hindlimb weakness is a prominent feature of this mouse model, we also analysed the tibialis anterior muscle. At P10, there was no evidence of denervation (Supplementary Fig. 2). This confirms that, although there is evidence of NMJ swelling in the P10, it is not more affected than the TVA muscle.

**Fig. 2:**
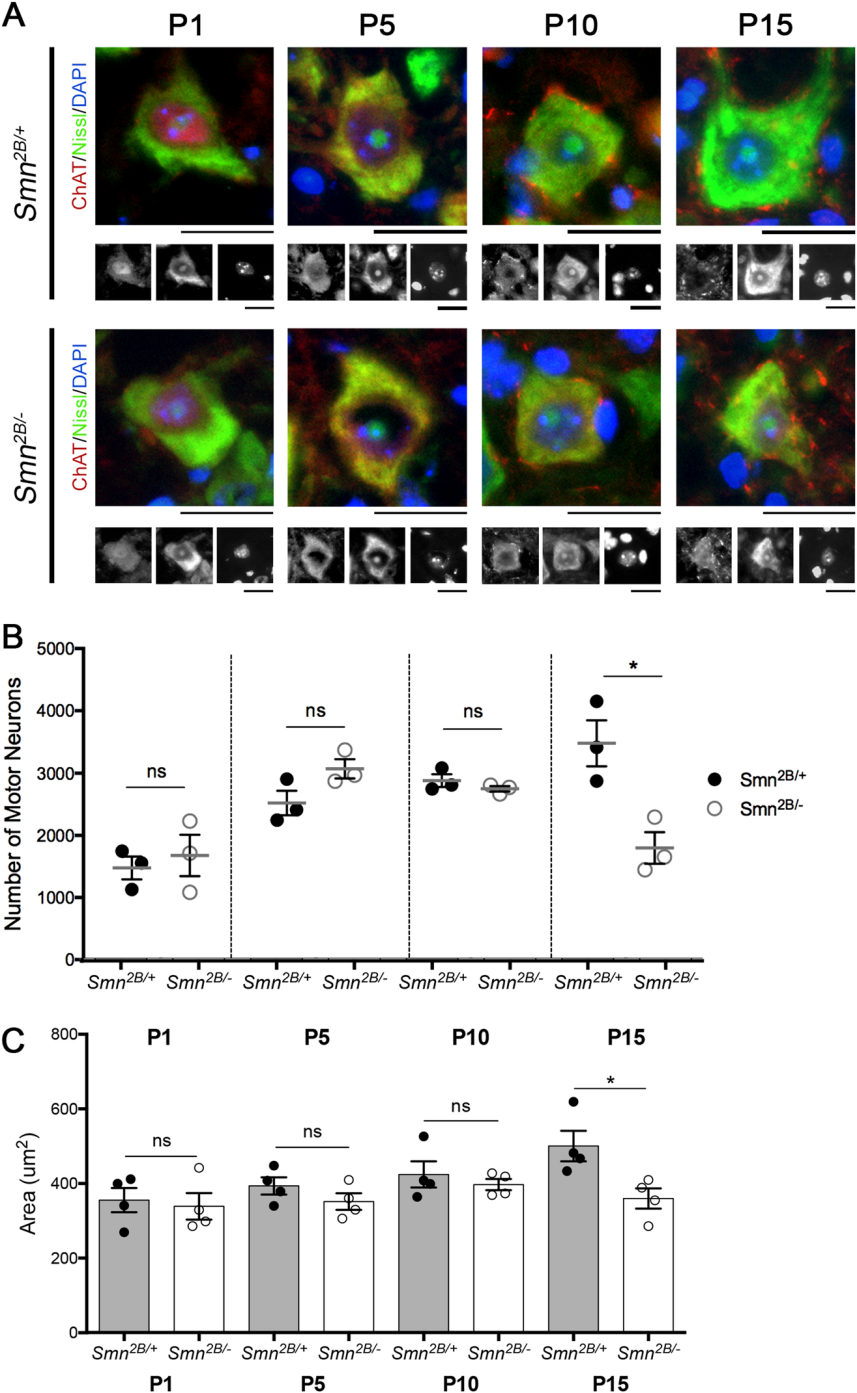
Alterations in MNCB number and size occur between P10 and P15 in the *Smn*^*2B/−*^ mouse model of SMA. **a** Representative images showing MNCBs from *Smn*^*2B/−*^ and *Smn*^*2B/+*^ control mice at P1, P5, P10 and P15. Note the comparable size difference between *Smn*^*2B/−*^ and *Smn*^*2B/+*^ mice at P15. The grayscale images shown below each coloured image are the separate channels and show, from left to right, ChAT, Nissl and DAPI staining. Scale bar = 20 µm. **b** The graph shows the absolute number of MNCBs counted in the ventral horn of the thoracic spinal cord of *Smn*^*2B/−*^ and control mice. At P1, P5 and P10 there is no significant difference between *Smn*^*2B/−*^ and *Smn*^*2B/+*^ pairs. At P15 there is a significant decrease in the number of MNCBs in *Smn*^*2B/−*^ mice compared to *Smn*^*2B/+*^ controls (by unpaired student's *t*-test, *p < 0.05, *n* = 3 mice per genotype, per time point). **c** The graph (mean ± SEM) shows that at P1, P5 and P10 there is no significant difference in the average area of MNCBs in the ventral horn of the spinal cord of *Smn*^*2B/−*^ and *Smn*^*2B/+*^ mice. However, there is a significant decrease in the average area of MNCBs in *Smn*^*2B/−*^ mice compared to *Smn*^*2B/+*^ mice at P15 (by Unpaired *t*-test, ns > 0.05, *p < 0.05, *n* = 4 mice per genotype)

To investigate how pre-synaptic pathology is associated with a loss of motor neuron cell bodies, we quantified the number of motor neurons in the thoracic spinal cord. The first decrease in motor neuron cell body number could be detected at P15, where a 48.4% decrease in motor neuron number was observed (Fig. 2b). Prior to this time point, there was no decrease in the number of motor neurons and indeed, an intriguing trend towards an increase in the number of motor neurons at P5, in the *Smn*^*2B/−*^ model compared to controls. There was also a significant decrease in motor neuron cell body area at P15, with no observable difference at P10 or younger (Fig. 2c).

Together with the data above, this demonstrates that NMJ pathology, which was first observed at P10, precedes motor neuron cell body shrinkage and loss, which was first observed at P15. This would suggest that motor neuron pathology does indeed progress in a distal to proximal manner, with NMJ loss preceding motor neuron cell body loss.

### NMJ pathology coincides with an increase in P53 related transcripts at the cell body

The data above indicate that NMJ pathology starts between P5 and P10. Previous work has shown that in this mouse model, at P10 there is an increase in transcripts associated with the P53 signalling pathway, including *Fas*, *Pmaip* and *Cdkn1a*^8^. Given the known links between the P53 signalling pathway and the onset of apoptosis, we have previously hypothesised that the increase in these transcripts was associated with the onset of motor neuron cell death. As we have now also observed that NMJ pathology first appears around the same time, it is possible that NMJ pathology is a consequence of P53 pathway activation at the cell body. We were therefore keen to explore the temporal relationship between an increase in transcripts associated with the P53 signalling pathway, and the onset of NMJ pathology.

**Fig. 3:**
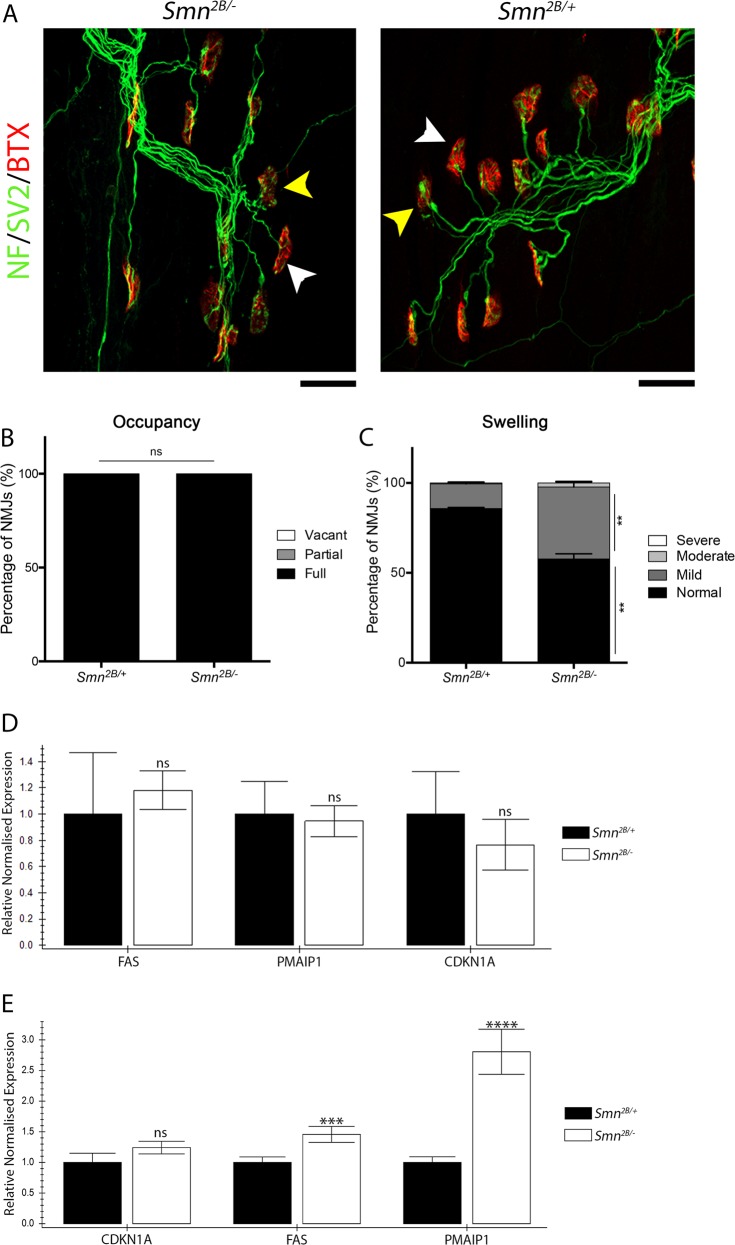
Activation of the P53 signalling pathway coincides with the onset of NMJ pathology. **a** Representative images of NMJs in the TVA of P7 *Smn*^*2B/−*^ and *Smn*^*2B/+*^ mice. White arrowheads show stage 1 swelling and yellow arrowheads show stage 2 pre-synaptic swelling. Scale bar = 20 µm. **b** Bar chart (mean ± SEM) shows that at P7, 100% of endplates in the TVA of *Smn*^*2B/−*^ and *Smn*^*2B/+*^ mice were fully occupied by the pre-synaptic terminal of an axon. (Mann–Whitney-U test, ns > 0.05; *n* = 4 mice per genotype). **c** Bar chart (mean ± SEM) compares the stages of pre-synaptic swelling in *Smn*^*2B/−*^ mice compared to *Smn*^*2B/+*^ controls. There is a significant decrease in the percentage of pre-synaptic terminals with stage 1 swelling and increase in stage 2 swelling in the TVA of *Smn*^*2B/−*^ mice compared to *Smn*^*2B/+*^ controls at P7. (Mann Whitney-U test, **p < 0.01; *n* = 4 mice per genotype). **d** The bar chart (mean ± SEM) shows that there is no significant difference in the expression of transcripts involved in the P53 signalling pathway in the thoracic spinal cord of *Smn*^*2B/−*^ mice compared to *Smn*^*2B/+*^ mice at P5 (by Mann–Whitney-U test; ns > 0.05; *n* = 4 per genotype). **e** The bar chart (mean ± SEM) shows that there is a significant increase in the expression of transcripts involved in the P53 signalling pathway, specifically in Cdkn1A and Pmaip1, in the thoracic spinal cord of *Smn*^*2B/−*^ mice compared to *Smn*^*2B/+*^ mice at P7 (by Mann–Whitney-U test; ns > 0.05; *n* = 4 per genotype)

We first aimed to further narrow down the time point at which NMJ pathology could be first seen. We therefore analysed NMJs in the TVA muscle from P7 mice (Fig. 3a–c). This revealed no evidence of denervation, but a significant increase in NMJs with mild pre-synaptic swelling. From this, we conclude denervation starts between P7 and P10 and is preceded by pre-synaptic swelling, which starts between P5 and P7.

Previous work has shown the transcripts *Fas*, *Pmaip* and *Cdkn1a* are significantly increased at P10 in this mouse model^8^. We therefore analysed spinal cords at P5 and P7. This revealed no increase in transcripts at P5, but a significant increase in *Fas* and *Pmaip* at P7 (Fig. 3d, e). Together this data shows a remarkable temporal correlation between the onset of NMJ swelling and the increase in transcripts associated with the P53 signalling pathway.

### Inhibition of the P53 signalling pathway reduces NMJ loss without affecting phenotype of the *Smn*^*2B/−*^ mouse model

Thus far we show that NMJ pathology precedes cell body loss, but coincides with the increase in transcripts associated with the P53 signalling pathway. To investigate the relationship between P53 pathway activation and NMJ pathology, we aimed to inhibit the P53 signalling pathway by generating an inducible P53 knockout mouse on an SMA model background. We generated this model by introducing a P53 gene flanked by loxP sites (P53^fl/fl^) as well as cre-recombinase under the control of the oestrogen receptor promoter (CAG-Cre) onto the *Smn*^*2B/−*^ background. The resultant *Smn*^*2B/−*^; *P53*^*fl/fl*^; *CAG-Cre* mice are equivalent to the original *Smn*^*2B/−*^ mouse model, however upon the administration of tamoxifen, the Cre-recombinase causes the recombination of the P53 gene to render it non-functional.

**Fig. 4:**
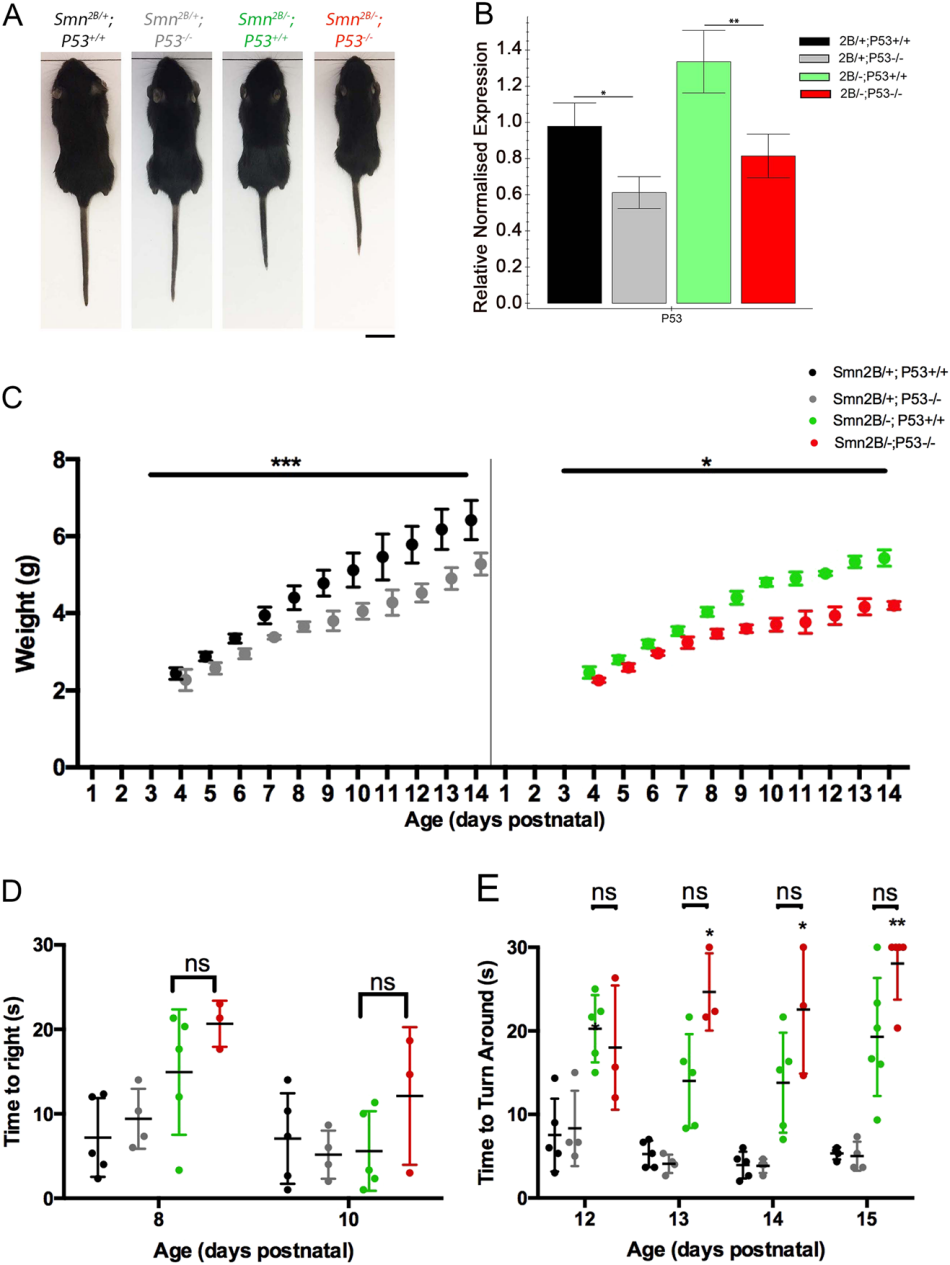
Reduction in P53 levels does not ameliorate the phenotype of the Smn^2B/−^ mouse model. **a** Representative images of mice with the various genotypes within experimental litters at P15. Scale bar = 1 cm. **b** Bar chart (mean ± SEM) shows that there is a significant decrease in P53 at the transcript level in the spinal cord of *Smn*^*2B/+*^; *P53*^*−/−*^ mice compared to *Smn*^*2B/+*^; *P53*^*+/+*^ mice and in *Smn*^*2B/−*^; *P53*^*−/−*^ mice compared to *Smn*^*2B/−*^; *P53*^*+/+*^ mice (by Mann–Whitney-U test, *p < 0.05, **p < 0.001; *n* = 3 for *Smn*^*2B/−*^; *P53*^*+/+*^, *n* = 4 for all other genotypes). **c** The graph (mean ± SEM) shows body weight in *Smn*^*2B/+*^; *P53*^*+/+*^ and *Smn*^*2B/+*^; *P53*^*−/−*^ on the left and *Smn*^*2B/−*^; *P53*^*+/+*^ and *Smn*^*2B/−*^; *P53*^*−/−*^ on the right. There is a significant reduction of weight in mice that are *P53*^*−/−*^ in both *Smn*^*2B/+*^ and *Smn*^*2B/−*^ groups, compared to *P53*^*+/+*^ mice. Two-way ANOVA with multiple comparison *P < 0.05; *P < 0.001 (**d**, **e**) Graphs (mean ± SEM, with individual data points showing mean of three trials for individual animals) shows performance in the time to right test (**d**) and the time to turn around test (**e**) for *Smn*^*2B/+*^; *P53*^*+/+*^, *Smn*^*2B/−*^; *P53*^*+/+*^, *Smn*^*2B/+*^; *P53*^*−/−*^ and *Smn*^*2B/−*^; *P53*^*−/−*^ at P8 and P10 (**d**) and between P12 and P15 (**e**). In both tests, at all-time points, there is no significant difference in motor performance between *Smn*^*2B/−*^; *P53*^*−/−*^ and *Smn*^*2B/−*^; *P53*^*+/+*^ mice. At P13, 14 and 15, *Smn*^*2B/−*^; *P53*^*−/−*^ displayed a significantly longer time to turn around than *Smn*^*2B/+*^; *P53*^*−/−*^ controls. (Kruskal Wallis with Dunn’s multiple comparison test; ns > 0.05, *p < 0.05, **p < 0.01, *n* = 5 for *Smn*^*2B/+*^; *P53*^*+/+*^ and *Smn*^*2B/−*^; *P53*^*+/+*^; *n* = 4 for *Smn*^*2B/+*^; *P53*^*−/−*^; *n* = 3 for *Smn*^*2B/−*^; *P53*^*−/−*^ for all time points except P15 where *n* = 4 for *Smn*^*2B/+*^; *P53*^*+/+*^ and *Smn*^*2B/+*^; *P53*^*−/−*^; *n* = 6 for *Smn*^*2B/−*^; *P53*^*+/+*^ and *n* = 5 for *Smn*^*2B/−*^; *P53*^*−/−*^

For these experiments we have four genotypes of mice: *Smn*^*2B/+*^ control mice who have the *P53*^*fl/fl*^ gene but do not carry CAG-Cre (*Smn*^*2B/+*^; *P53*^*+/+*^); *Smn*^*2B/−*^ mice who have the *P53*^*fl/fl*^ gene but do not carry CAG-Cre (*Smn*^*2B/−*^; P53^+/+^); *Smn*^*2B/+*^control mice who have the *P53*^*fl/fl*^ gene and CAG-Cre (Smn^2B/+^; P53^−/−^); and *Smn*^*2B/−*^ mice who have the *P53*^*fl/fl*^ gene and CAG-Cre (*Smn*^*2B/−*^; P53^−/−^) (Fig. 4a). Upon administration of tamoxifen, the P53 gene in only the latter two groups of mice will undergo recombination and essentially be ‘knocked out’. Tamoxifen (75 mg/kg) was administered at P4 and P5. This time point was selected as it was prior to any evidence of P53 pathway activation in the spinal cord. Quantitative PCR results on cDNA from spinal cord from P15 mice revealed P53 levels were reduced by around 50% in both *Smn*^*2B/+*^; *P53*^*−/−*^ and *Smn*^*2B/−*^; *P53*^*−/−*^ mice compared to their respective controls (Fig. 4b).

To determine the effect of P53 knockout on the phenotype of the *Smn*^*2B/−*^ mouse, weight and motor performance were assessed daily following administration of tamoxifen at P4 and P5. Knockout of P53 did not improve the phenotype of *Smn*^*2B/−*^ mice (Fig. 4c–e). Indeed, both groups with reduced P53 levels displayed a small but significant decrease in body weight. There was a trend towards poorer performance in the time to right test for both *Smn*^*2B/−*^ groups at P8, but there was no significant difference between *Smn*^*2B/−*^; *P53*^*+/+*^ and *Smn*^*2B/−*^; *P53*^*−/−*^ at either P8 or P10. Furthermore, in the ‘turn around’ test, although *Smn*^*2B/−*^*;P53*^*−/−*^ mice performed significantly worse at P13, 14 and 15 compared to *Smn*^*2B/+*^; *P53*^*−/−*^ controls, there was no significant difference between *Smn*^*2B/−*^; *P53*^*+/+*^ and *Smn*^*2B/−*^; *P53*^*−/−*^ mice. This data demonstrate that reduction of P53 confers no benefit to the motor performance of *Smn*^*2B/−*^ mice, and if anything, makes it worse.

**Fig. 5:**
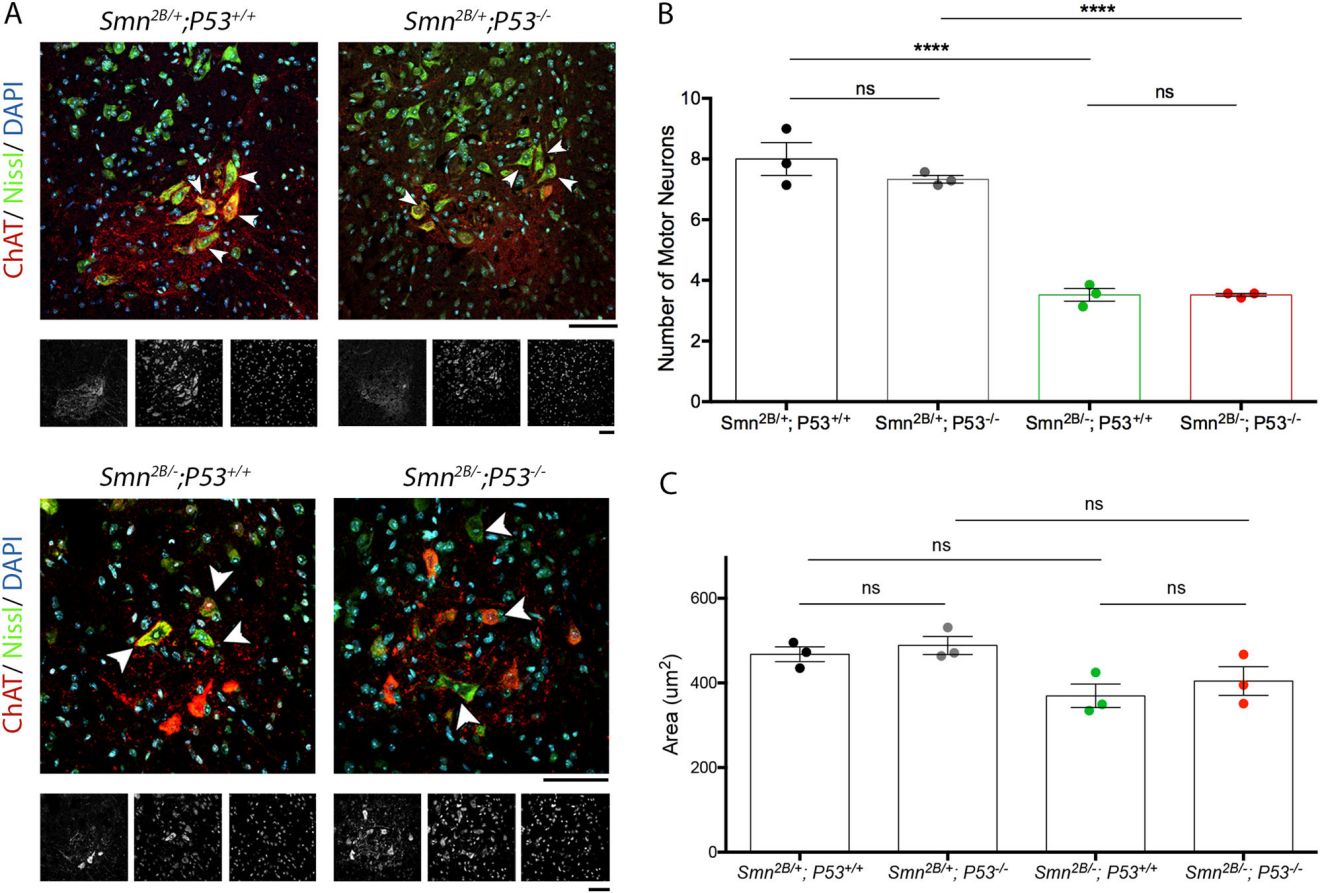
Reducing P53 levels in *Smn*^*2B/−*^ mice does not alter the number or size of MNCBs in the ventral horn of the spinal cord at P15. **a** Representative images showing MNCBs in the ventral horn of the spinal cord at P15of *Smn*^*2B/+*^; *P53*^*+/+*^ and *Smn*^*2B/+*^; *P53*^*−/−*^ mice and *Smn*^*2B/−*^; *P53*^*+/+*^ and *Smn*^*2B/−*^; *P53*^*−/−*^ mice. White arrowheads point out MNCBs. The grayscale images shown below each coloured image are the sperate channels and show, from left to right, ChAT, Nissl and DAPI staining. Scale bar = 40 μm. **b** The graph (mean ± SEM) shows that when P53 is reduced there is no significant difference in the number of MNCBs in the ventral horn of both *Smn*^*2B/+*^ and *Smn*^*2B/−*^ spinal cords at P15. There is a significant difference in the loss of MNCBs in *Smn*^*2B/−*^ mice compared to *Smn*^*2B/+*^ mice (by one-way ANOVA; ns > 0.05, ****p < 0.001; *n* = 3 mice per genotype). **c** The graph (mean ± SEM) shows that when P53 is reduced there is no significant difference in the area of MNCBs in the ventral horn of both *Smn*^*2B/+*^ and *Smn*^*2B/−*^ spinal cords (by one-way ANOVA; ns > 0.05; *n* = 3 mice per genotype)

We next assessed the impact of reduced P53 levels on motor neuron cell body loss. Reduced P53 levels did not affect motor neuron cell body loss or shrinkage. Quantification of the number of motor neurons in the T4–T8 segments of the spinal cord at P15 revealed a loss of 55.2 ± 9.6% and 52.0 ± 1.7% in *Smn*^*2B/−*^; *P53*^*+/+*^ and *Smn*^*2B/−*^; *P53*^*−/−*^ compared to their respective controls (Fig. 5b). There was no significant difference between *Smn*^*2B/−*^; *P53*^*+/+*^and *Smn*^*2B/−*^; *P53*^*−/−*^ mice. There was a trend for motor neurons from *Smn*^*2B/−*^; *P53*^*+/+*^ and *Smn*^*2B/−*^; *P53*^*−/−*^ mice to be smaller compared to their respective controls but again there was no significant difference between *Smn*^*2B/−*^; *P53*^*+/+*^ and *Smn*^*2B/−*^; *P53*^*−/−*^ mice (Fig. 5c). Overall, in the *Smn*^*2B/−*^ mouse model, reduced P53 levels do not appear to reduce motor neuron cell body loss or shrinkage.

**Fig. 6:**
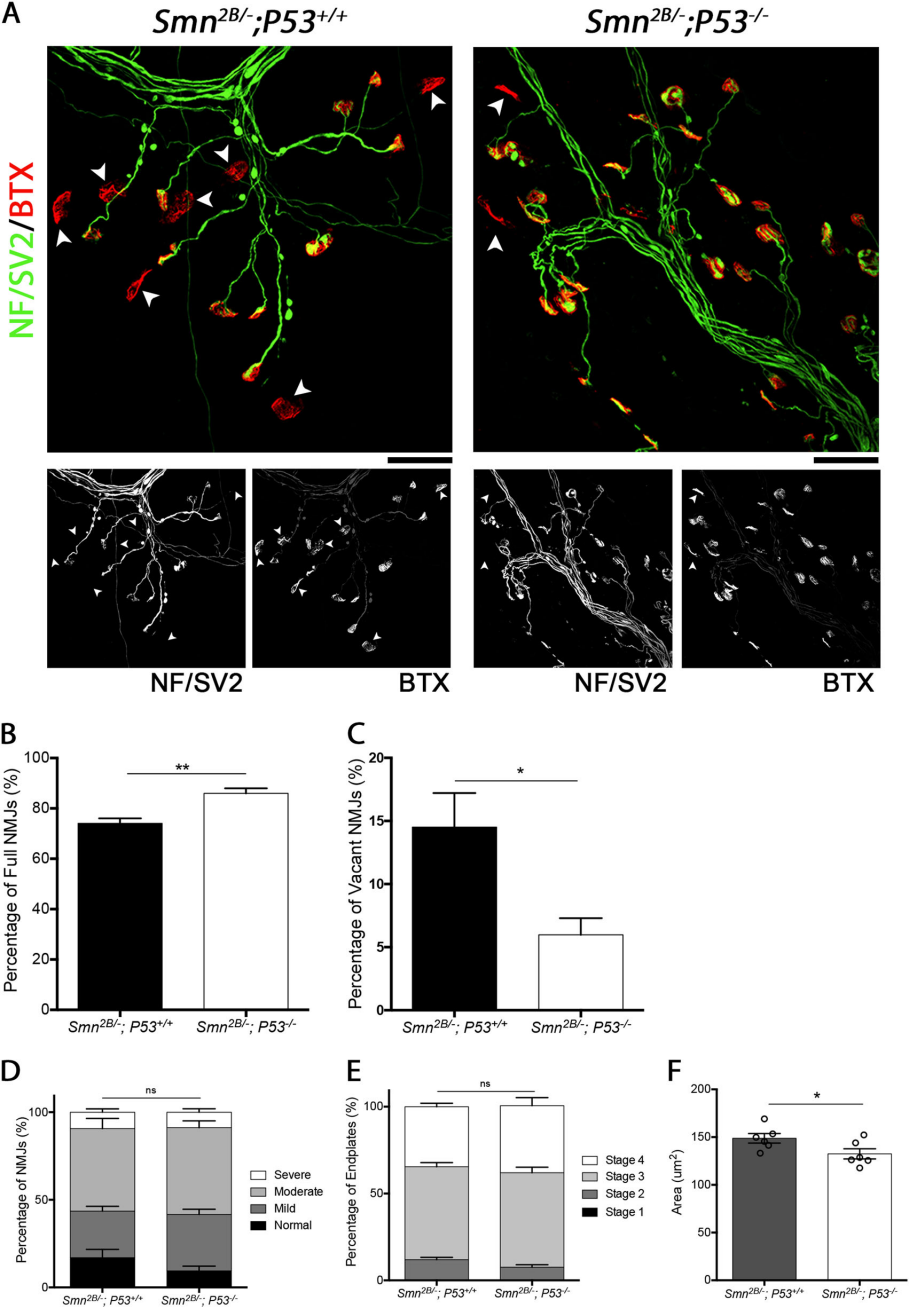
Reduction in P53 reduces NMJ loss but does not affect other morphological signs of degeneration. **a** Representative images showing NMJs from the TVA of *Smn*^*2B/−*^; *P53*^*+/+*^ and *Smn*^*2B/−*^; *P53*^*−/−*^ mice. Note that the white arrowheads point out vacant endplates. Scale bar = 40 μm. **b** The graph (mean ± SEM) shows that a reduction in P53 decreases denervation in the TVA muscle, with *Smn*^*2B/−*^; *P53*^*−/−*^ mice showing a significant increase in the percentage of fully occupied endplates compared to *Smn*^*2B/−*^; *P53*^*+/+*^ mice (by Mann–Whitney-U test; **p < 0.01; *n* = 6 muscles per genotype). **c** In addition, this graph (mean ± SEM) shows a significant decrease in the percentage of vacant endplates in *Smn*^*2B/−*^; *P53*^*−/−*^ mice compared to *Smn*^*2B/−*^; *P53*^*+/+*^ mice (by Mann–Whitney-U test; *p < 0.05; *n* = 6 muscles per genotype). **d** The graph (mean ± SEM) shows that there is no significant difference in the level of pre-synaptic swelling in the TVA of *Smn*^*2B/−*^; *P53*^*+/+*^ and *Smn*^*2B/−*^*; P53*^*−/−*^ mice (by Mann–Whitney-U test; ns > 0.05; *n* = 6 muscles per genotype). **e** The graph (mean ± SEM) shows that there is no significant difference in the maturity of endplates in the TVA of *Smn*^*2B/−*^; *P53*^*+/+*^ and *Smn*^*2B/−*^; *P53*^*−/−*^ mice (by Mann–Whitney-U test; ns > 0.05; *n* = 6 muscles per genotype). **f** The graph (mean ± SEM) shows that there is a small but significant decrease in the area of endplates in the TVA of *Smn*^*2B/−*^; *P53*^*+/+*^ and *Smn*^*2B/−*^; *P53*^*−/−*^ mice (by Unpaired T-test; *p < 0.05; *n* = 6 muscles per genotype)

Finally, we assessed the effect of reduced P53 levels of NMJ pathology. This revealed that reduced P53 levels decreased NMJ loss (Fig. 6). Quantification of the percentage of fully occupied endplates at P15 in *Smn*^*2B/−*^; *P53*^*−/−*^ mice revealed a significant increase (Fig. 6b). This was accompanied by a significant decrease in the percentage of vacant endplates (Fig. 6c). Interestingly, examination of pre-synaptic swelling, endplate maturation and endplate area revealed no improvement in *Smn*^*2B/−*^; *P53*^*−/−*^ compared to *Smn*^*2B/−*^; *P53*^*+/+*^ (Fig. 6d–f). Together this data suggest that NMJ loss is a consequence of P53 pathway activation, and dissociates NMJ loss from other markers of pathology at the NMJ, which are P53 independent.

## Discussion

Here we have shown that pathology at the NMJ precedes loss or shrinkage of motor neurons at the level of the cell body. A loss of NMJs was first observed at P10, whereas motor neuron loss was not observed until P15. This first evidence of NMJ swelling appears to coincide with an increase in transcripts associated with the P53 signalling pathway in the spinal cord. Importantly, post-natal ‘knockout’ of P53 led to a reduction in NMJ loss, without affecting other aspects of NMJ pathology, motor neuron loss or affecting the phenotype of the mouse. Together the work provides a detailed temporal description of pathology within the motor units of an SMA mouse model, and demonstrates that NMJ loss is a P53-dependent process. This work supports the role for P53 as an effector of synaptic and axonal degeneration in a die-back neuropathy.

### Temporal and mechanistic dissociation of motor unit pathology in SMA

The protracted lifespan of the *Smn*^*2B/−*^ mouse, compared to the majority of other commonly used mouse models, has allowed us to examine the evolution of different aspects of motor unit pathology, and revealed a temporal dissociation between them. Motor neuron loss and shrinkage is one of the last detectable morphological abnormalities and evident only after symptom onset. Swelling of the pre-synaptic terminal preceded NMJ loss and post-synaptic changes, and also preceded the emergence of symptoms. This is in agreement with studies on the SOD1^G93A^ mouse model of adult onset motor neuron disease in which pre-synaptic defects were shown to coincide with symptom onset, and motor neuron cell body loss was observed as a late event^31^. Swelling appeared to coincide with the earliest time point that increased levels of *Fas*, and *Pmaip* can be detected. Given the known links of these transcripts with the P53 signalling pathway, this suggests that there is a correlation between the onset of NMJ abnormalities and P53 pathway activation. Together this data suggest that, with regards to motor unit pathology, the first detectable abnormality is swelling of NMJs, which coincides with transcriptional changes at the cell body. This is then followed by denervation, then by post-synaptic shrinkage and loss and shrinkage of the motor neuron cell body.

The reduction in P53 levels allows us to more carefully dissect the interdependency of these early events. Reduced P53 levels had no effect on swelling, but did reduce denervation. This shows that pre-synaptic swelling is a P53-independent event, whilst denervation is a P53-dependant event. Thereby, whilst the pathology present at the NMJ is not dependent upon the P53 pathway, the actual process of pre-synaptic withdrawal is.

### A role for P53 in mediating synaptic and axonal degeneration

The data presented here demonstrate that knockout of P53 can reduce NMJ loss, and therefore suggest that axon and synaptic degeneration in SMA is a P53-dependant process. P53 has been identified in synaptosomes of cortical neurons^26^. Synaptosomes from P53 knockout mice displayed reduced levels of reactive oxygen species and preserved mitochondrial membrane potential following exposure to the topoisomerase-II inhibitor etoposide^26^. Furthermore, mitochondria in isolated synaptosomes from P53 knockout mice showed greater resistance to excitotoxic or oxidative insults, and inhibition of P53 decreased synapse loss in hippocampal neuron cultures following treatment with etoposide. This clearly shows that P53 can have a local role in mediating synapse loss and mitochondrial dysfunction. An increase in P53 phosphorylation has also been observed following trophic withdrawal, a stimulus known to result in axon pruning^32^. How then, might P53 mediate the removal of axonal and synaptic compartments of the cell? A significant body of work has implicated pro-apoptotic proteins and pathways in the process of axon degeneration^33^. Interestingly, this includes developmental axon pruning, which demonstrates that the activation of apoptotic cascades can be compartment specific and that mediating removal of axonal and synaptic parts of the neuron allow the motor neuron to persist. Indeed, it has been suggested that as P53 has the ability to regulate both pro- and anti-apoptotic transcripts, it can serve as a key regulator for compartmental degeneration in neurons^32,34^. Further work is clearly required to identify the molecular effectors of P53-dependant synaptic degeneration in SMA, and determine whether P53 is an important mediator of axonal and synaptic degeneration in other neurodegenerative contexts.

### P53 independent motor neuron loss in SMA

An upregulation of transcripts associated with the P53 signalling pathway has been consistently observed in response to reduced Smn levels^8,21–24^. Due to the well-established links between P53 and caspase mediated apoptosis, this has led to the suggestion that motor neurons in SMA are dying by a P53 mediated form of apoptosis. In this canonical pathway, an increase in cell stress, brought about by a variety of triggers, causes the phosphorylation and activation of P53, which in turn act as a transcription factor for a number of pro-apoptotic transcripts including *Bax*, *Bak* and *Pmaip*. These factors cause an increase in the permeability of the mitochondrial membrane, resulting in a release of pro-apoptotic factors, leading to the activation of effector caspases. This includes caspase 3, which mediates the cleavage of cellular components and is essential for the chromatin condensation and DNA fragmentation, all of which are morphological hallmarks of apoptosis. The data presented here demonstrate that inhibition of P53 does not reduce motor neuron loss, suggesting that motor neuron loss in this mouse model is P53 independent. Although this is in contrast to recent reports showing P53 inhibition could reduce motor neuron loss in the *Smn*^*−/−*^*; SMN2; SMNΔ7* mouse model of SMA, this finding is in agreement with previous work showing knockout of P53 has no beneficial effect on a mouse model of SMA^9,35^. There are also interesting parallels to work on the SOD^G93A^ mouse model of ALS, showing knockout of P53 has no effect upon motor neuron loss^36^, despite the observation that P53 levels are elevated in postmortem tissue, cellular models and mouse models of ALS^16,17,19,37–40^. Whilst this could be due to a degree of redundancy between P53 and related pathways, it does demonstrate that motor neurons in SMA can die by a P53 independent mechanism.

Studies detailing apoptotic motor neurons in SMA are notably lacking. Although some have reported features consistent with apoptosis in postmortem and cellular and animal models, work using immunohistochemical approaches to label apoptotic revealed no difference between SMA and control patients in either fetal or newborn individuals with SMA^20,35,41,42^. However, knockout of the pro-apoptotic protein Bax in a mouse model of SMA did reduce motor neuron loss^43^. More work is required to define whether motor neurons in SMA are degenerating via a caspase dependant apoptosis, and investigate P53 independent up-stream regulators of this process. Identifying this mechanism will be of fundamental importance if we are to gain a thorough understanding of motor unit pathogenesis in SMA.

## Materials and methods

### Mouse maintenance

*Smn*^*2B/2B*^ (originating from the Kothary Laboratory, Ottawa Hospital Research Institute) mice were crossed with *Smn*^*+/−*^ (Strain: 10921, Jackson Laboratories) to produce *Smn*^*2B/−*^ experimental mice and *Smn*^*2B/+*^ controls. *Smn*^*+/−*^ mice were crossed with mice carrying the *P53*^*fl/fl*^ and *CAG-Cre* alleles (generously donated by Dr Luke Boulter, Kevin Myant and You-Ying Chou, University of Edinburgh) to obtain *Smn*^*+/−*^; *P53*^*fl/fl*^; *CAG-Cre* mice. *Smn*^*2B/2B*^ mice were interbred with mice carrying the *P53*^*fl/fl*^ allele to obtain *Smn*^*2B/2B*^; *P53*^*fl/fl*^ mice. *Smn*^*+/−*^; *P53*^*fl/fl*^; *CAG-Cre* and *Smn*^*2B/2B*^; *P53*^*fl/fl*^ mice were interbred to produce *Smn*^*2B/+*^; *P53*^*fl/fl*^; *CAG-Cre, Smn*^*2B/+*^; *P53*^*fl/fl*^*, Smn*^*2B/−*^; *P53*^*fl/fl*^; *CAG-Cre and Smn*^*2B/−*^; *P53*^*fl/fl*^ mice. All genotyping was performed using standard PCR protocols. Homozygosity for the P53^fl^ allele was confirmed using q-PCR. All mice were maintained at the University of Edinburgh animal facilities on a C57Bl6 background. Mice aged over P10 were sacrificed by overdose of inhalation anaesthetic or a rising CO_2_ and death was confirmed by exsanguination from the carotid artery. Mice under P10 were decapitated under terminal anaesthesia and death was confirmed by exsanguination from the carotid artery. Endstage of disease was defined by over 10% loss of weight over a period of 48 h. Since no phenotypic differences have been noted between male and female mice, experiments were not controlled for gender. All experiments were performed in accordance with the UK Home Office.

### Tamoxifen

Tamoxifen (Sigma-Aldrich) was dissolved in corn oil to a concentration of 20 mg/ml. Tamoxifen solution was administered to litters of mice at P4 and P5 using oral gavage at a dose of 75 mg/kg.

### Phenotypic analysis

The ‘time to right’ test was carried out on mice at P8 and P10. Mice were placed on their back and the time it took for them to right themselves (turn to be on all four paws) was noted, with a maximum of 30 sec and an average of three attempts.

The ‘turn around test’ was used on mice daily aged over P12. This test involved placing mice, head down on a gridded cage top that was at a 45^o^ angle. The time it took for mice to turn 180^o^ to face up was noted, with a maximum of 30 sec and an average of three attempts.

### Neuromuscular junction labelling and quantification

Muscles were immediately dissected from recently sacrificed mice and fixed in 4% Paraformaldehyde (PFA; Electron Microscopy Science) in PBS for 15 min. Post-synaptic AChRs were labelled with α-bungarotoxin (BTX, 1:250; tetramethylrhodimine conjugate, Life Technologies) for 2 h. Muscles were permeabilised in 2% Triton X-100 in PBS for 30 min, then blocked in 4% bovine serum albumin (BSA)/1% Triton X-100 in PBS for 30 min before incubation overnight in primary antibodies [Neurofilament, 1:50 (NF; 2H3)—Developmental Studies Hybridoma Bank; synaptic vesicle protein 2, 1:100 (SV2)—Developmental Studies Hybridoma Bank] and visualised with secondary antibodies [AlexaFluor-488 rabbit anti-mouse at 1:250, Jackson]. Muscles were then whole-mounted in Mowiol (Sigma).

All images were quantified using a Leica inverted fluorescent microscope using single and dual wavelength filter sets allowing separate and simulations visualisation of red and green channels. The percentage of fully occupied endplates was determined by classifying each endplate in a given field of view either fully occupied (pre-synaptic terminal (SV2 and NF) completely overlies endplate (BTX)), partially occupied (pre-synaptic terminal only partially covers endplate (BTX)), or vacant (no pre-synaptic label overlies endplate). For pre-synaptic swelling, all NMJs were classified as having no swelling, mild (evidence of some axonal swelling and/or pre-synaptic swelling that does not obscure the endplate), moderate (evidence of clear axonal swelling and significant pre-synaptic swelling that is beginning to obscure the endplate) or severe (significant and obvious swelling along the length of the axon and severe swelling around the pre-synaptic terminal, which obscures the endplate). Endplate size was quantified using ImageJ software. Endplate maturation was quantified using a stage 1–4 categorisation, where stage 4 endplates are immature and have a plaque-like appearance, stage 3 endplates are developing from a plaque-like structure with folds visible on the plaque, stage 2 endplates are developing into a pretzel-like structure with folds and perforations visible and stage 1 endplates have a mature pretzel-like structure. At least three fields of view were analysed per muscle totalling >80 endplates per muscle. All Quantification was performed with researcher blind to genotype of material. All example images are z-stack projections and were obtained using a Nikon confocal microscope at ×40 magnification and images had pixel size of 1024 × 1024.

### Motor neuron immuno-fluorescence and quantification

Spinal cords were removed from recently sacrificed mice and fixed in 4% PFA (Electron Microscopy Science) for 4 h. They were immersed in 30% sucrose for 48 h prior to embedding in 50% OCT; 15% Sucrose in PBS mixture. For temporal analysis of *Smn*^*2B/−*^ mice, the ventral horn of thoracic (T1-T12) spinal cords were sectioned longitudinally at 10 µM and alternate sections were subjected to Nissl/ChAT labelling. For analysis of P53^fl/fl^ mice, thoracic (T4-T8) spinal cords were sectioned coronally at 10 µM and every tenth section was subjected to Nissl/ChAT labelling.

Sections were washed in PBS, permeabilised in 0.3% Triton X-100 in PBS for 30 min and blocked in 0.1% Power Block (100× , Biogenex Laboratories, HK085–5K) in PBS for 10 min. Sections were incubated in ChAT (Goat anti-choline acetyl-transferase; Merck Millipore; 1:100) primary antibody in 0.3% Triton X-100; 1% Bovine Serum Albumin (BSA) in PBS at 4 °C, for three nights. Secondary antibody (AlexaFluor 555 Donkey anti-Goat; Life Technologies; 1:250) was applied for 2 h at a dilution of 1:250 at room temperature. Sections were then stained with DAPI (Life Technologies; 1:1000) and fluorescent Nissl stain (NeuroTrace; Life Technologies: 1:100). Sections were mounted in Mowiol (Sigma).

For quantification of motor neuron number, the number of ChAT positive cells with a visible nucleolus were quantified in each section. For Fig. 2, this was done on longitudinal section, quantifying every second section. For Fig. 5, this was done on coronal section, for every 30th section. For motor neuron cell body area, images were captured on a standard fluorescent microscope (Leica DM8) and the perimeter of each cell was traced using ImageJ software. All data was assembled in Microsoft Excel and analysed using GraphPad Prism.

### qRT-PCR

RNA was extracted using a micro RNeasy Kit (Qiagen) and 1 µg of RNA was used to perform reverse transcriptase using the RT^2^ First Strand Kit (Qiagen). SYBR gene based Q-RT-PCR was performed using pre-optimised primers purchased from Qiagen. Amplification was performed using KAPA SYBR fast universal PCR mastermix as per manufacturer's instructions on a BioRad CFX connect real-time PCR detection system. Relative gene expression was calculated using the 2^−ΔΔcT^ formula^44^. Relative levels are expressed normalised to Actin (F: CCGTCAGGCAGCTCATAGCTCTTC; R: CTGAACCCTAAGGCCAACCGT), GusB (F: GGCTGGTGACCTACTGGATTT; R: TTGGCACTGGGAACCTGAAGT) and YWHAZ (F: TTGATCCCCAATGCTTCGC; R:: CAGCAACCTCGGCCAAGTAA).

## Supplementary information

Supplementary Figure 1.

Supplementary Figure 2.

Supplementary figure legends.

## References

[1] Lefebvre S (1995). Identification and characterization of a spinal muscular atrophy-determining gene. Cell.

[2] Rodrigues NR (1995). Deletions in the survival motor neuron gene on 5q13 in autosomal recessive spinal muscular atrophy. Hum. Mol. Genet..

[3] Bebee TW, Dominguez CE, Chandler DS (2012). Mouse models of SMA: tools for disease characterization and therapeutic development. Hum. Genet..

[4] Goulet BB, Kothary R, Parks RJ (2013). At the “junction” of spinal muscular atrophy pathogenesis: the role of neuromuscular junction dysfunction in SMA disease progression. Curr. Mol. Med..

[5] Tisdale S, Pellizzoni L (2015). Disease mechanisms and therapeutic approaches in spinal muscular atrophy. J. Neurosci..

[6] Murray LM (2008). Selective vulnerability of motor neurons and dissociation of pre- and post-synaptic pathology at the neuromuscular junction in mouse models of spinal muscular atrophy. Hum. Mol. Genet..

[7] Cifuentes-Diaz C (2002). Neurofilament accumulation at the motor endplate and lack of axonal sprouting in a spinal muscular atrophy mouse model. Hum. Mol. Genet..

[8] Murray LM, Beauvais A, Gibeault S, Courtney NL, Kothary R (2015). Transcriptional profiling of differentially vulnerable motor neurons at pre-symptomatic stage in the Smn (2b/−) mouse model of spinal muscular atrophy. Acta Neuropathol. Commun..

[9] Simon CM (2017). Converging mechanisms of p53 activation drive motor neuron degeneration in spinal muscular atrophy. Cell Rep..

[10] Chaudhary Ritu, Lal Ashish (2016). Long noncoding RNAs in the p53 network. Wiley Interdisciplinary Reviews: RNA.

[11] Marcel V, Catez F, Diaz JJ (2015). p53, a translational regulator: contribution to its tumour-suppressor activity. Oncogene.

[12] Mei Y, Wu M (2016). Noncoding RNAs regulating p53 and c-Myc signaling. Adv. Exp. Med. Biol..

[13] Marcel Virginie, Nguyen Van Long Flora, Diaz Jean-Jacques (2018). 40 Years of Research Put p53 in Translation. Cancers.

[14] Chang JR (2012). Role of p53 in neurodegenerative diseases. Neurodegener. Dis..

[15] de la Monte SM, Sohn YK, Wands JR (1997). Correlates of p53- and Fas (CD95)-mediated apoptosis in Alzheimer’s disease. J. Neurol. Sci..

[16] Martin LJ (2000). p53 is abnormally elevated and active in the CNS of patients with amyotrophic lateral sclerosis. Neurobiol. Dis..

[17] Ranganathan S, Bowser R (2010). p53 and cell cycle proteins participate in spinal motor neuron cell death in ALS. Open Pathol. J..

[18] Szybinska A, Lesniak W (2017). P53 dysfunction in neurodegenerative diseases - the cause or effect of pathological changes?. Aging Dis..

[19] Vogt MA (2018). TDP-43 induces p53-mediated cell death of cortical progenitors and immature neurons. Sci. Rep..

[20] Simic G (2000). Ultrastructural analysis and TUNEL demonstrate motor neuron apoptosis in Werdnig-Hoffmann disease. J. Neuropathol. Exp. Neurol..

[21] Baumer D (2009). Alternative splicing events are a late feature of pathology in a mouse model of spinal muscular atrophy. PLoS Genet..

[22] Jangi M (2017). SMN deficiency in severe models of spinal muscular atrophy causes widespread intron retention and DNA damage. Proc. Natl Acad. Sci. USA.

[23] Staropoli JF (2015). Rescue of gene-expression changes in an induced mouse model of spinal muscular atrophy by an antisense oligonucleotide that promotes inclusion of SMN2 exon 7. Genomics.

[24] Zhang Z (2008). SMN deficiency causes tissue-specific perturbations in the repertoire of snRNAs and widespread defects in splicing. Cell.

[25] Young PJ (2002). A direct interaction between the survival motor neuron protein and p53 and its relationship to spinal muscular atrophy. J. Biol. Chem..

[26] Gilman CP (2003). p53 is present in synapses where it mediates mitochondrial dysfunction and synaptic degeneration in response to DNA damage, and oxidative and excitotoxic insults. Neuromolecular Med..

[27] Lau D, Bading H (2009). Synaptic activity-mediated suppression of p53 and induction of nuclear calcium-regulated neuroprotective genes promote survival through inhibition of mitochondrial permeability transition. J. Neurosci..

[28] Merlo P (2014). p53 prevents neurodegeneration by regulating synaptic genes. Proc. Natl Acad. Sci. USA.

[29] Bowerman M, Murray LM, Beauvais A, Pinheiro B, Kothary R (2012). A critical smn threshold in mice dictates onset of an intermediate spinal muscular atrophy phenotype associated with a distinct neuromuscular junction pathology. Neuromuscul. Disord..

[30] Thomson SR (2012). Morphological characteristics of motor neurons do not determine their relative susceptibility to degeneration in a mouse model of severe spinal muscular atrophy. PLoS ONE.

[31] Gould TW (2006). Complete dissociation of motor neuron death from motor dysfunction by Bax deletion in a mouse model of ALS. J. Neurosci..

[32] Maor-Nof M (2016). Axonal degeneration is regulated by a transcriptional program that coordinates expression of pro- and anti-degenerative factors. Neuron.

[33] Geden MJ, Deshmukh M (2016). Axon degeneration: context defines distinct pathways. Curr. Opin. Neurobiol..

[34] Lassus P, Ferlin M, Piette J, Hibner U (1996). Anti-apoptotic activity of low levels of wild-type p53. EMBO J..

[35] Tsai MS, Chiu YT, Wang SH, Hsieh-Li HM, Li H (2006). Abolishing Trp53-dependent apoptosis does not benefit spinal muscular atrophy model mice. Eur. J. Hum. Genet..

[36] Kuntz Ct, Kinoshita Y, Beal MF, Donehower LA, Morrison RS (2000). Absence of p53: no effect in a transgenic mouse model of familial amyotrophic lateral sclerosis. Exp. Neurol..

[37] Barbosa LF (2010). Increased SOD1 association with chromatin, DNA damage, p53 activation, and apoptosis in a cellular model of SOD1-linked ALS. Biochim. Biophys. Acta.

[38] de la Monte SM, Sohn YK, Ganju N, Wands JR (1998). P53- and CD95-associated apoptosis in neurodegenerative diseases. Lab. Invest..

[39] Eve DJ, Dennis JS, Citron BA (2007). Transcription factor p53 in degenerating spinal cords. Brain Res..

[40] Wang J (2014). TDP-43 interaction with the intracellular domain of amyloid precursor protein induces p53-associated apoptosis. Neurosci. Lett..

[41] Ito Y, Shibata N, Saito K, Kobayashi M, Osawa M (2011). New insights into the pathogenesis of spinal muscular atrophy. Brain Dev..

[42] Piras A (2017). Inhibition of autophagy delays motoneuron degeneration and extends lifespan in a mouse model of spinal muscular atrophy. Cell Death. Dis..

[43] Tsai MS (2006). Abolishing Bax-dependent apoptosis shows beneficial effects on spinal muscular atrophy model mice. Mol. Ther..

[44] Livak KJ, Schmittgen TD (2001). Analysis of relative gene expression data using real-time quantitative PCR and the 2(-Delta Delta C(T)) method. Methods.

