# Supplementary figures and images for: Reduced P53 levels ameliorate neuromuscular junction loss without affecting motor neuron pathology in a mouse model of spinal muscular atrophy
Source: Cell Death Dis. 2019 Jul 4;10(7):515. doi: 10.1038/s41419-019-1727-6 (PMC6609617; doi:10.1038/s41419-019-1727-6)

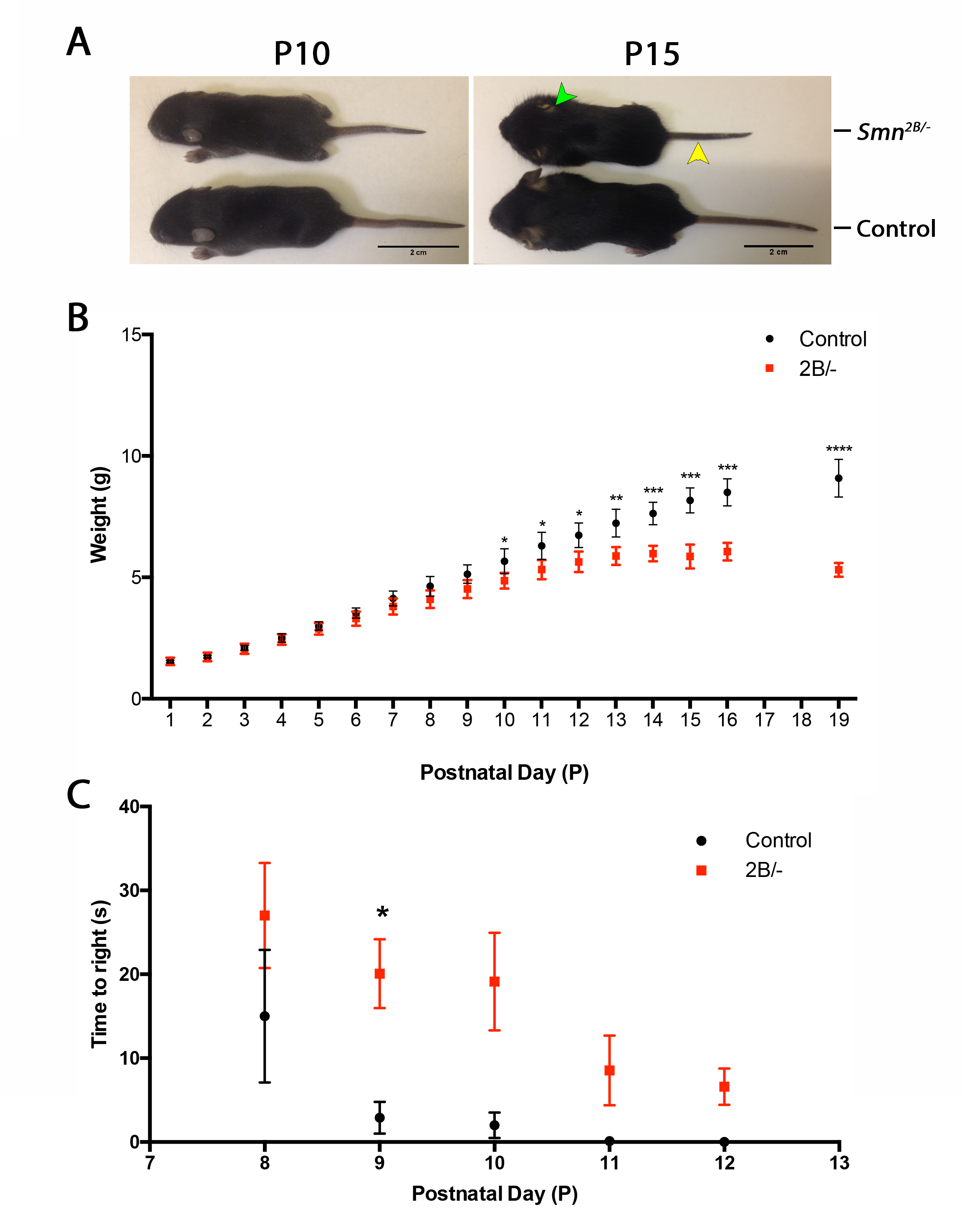

Supplement: Supplementary file 1 — Supplementary Figure 1. [file 41419_2019_1727_MOESM1_ESM.tif]

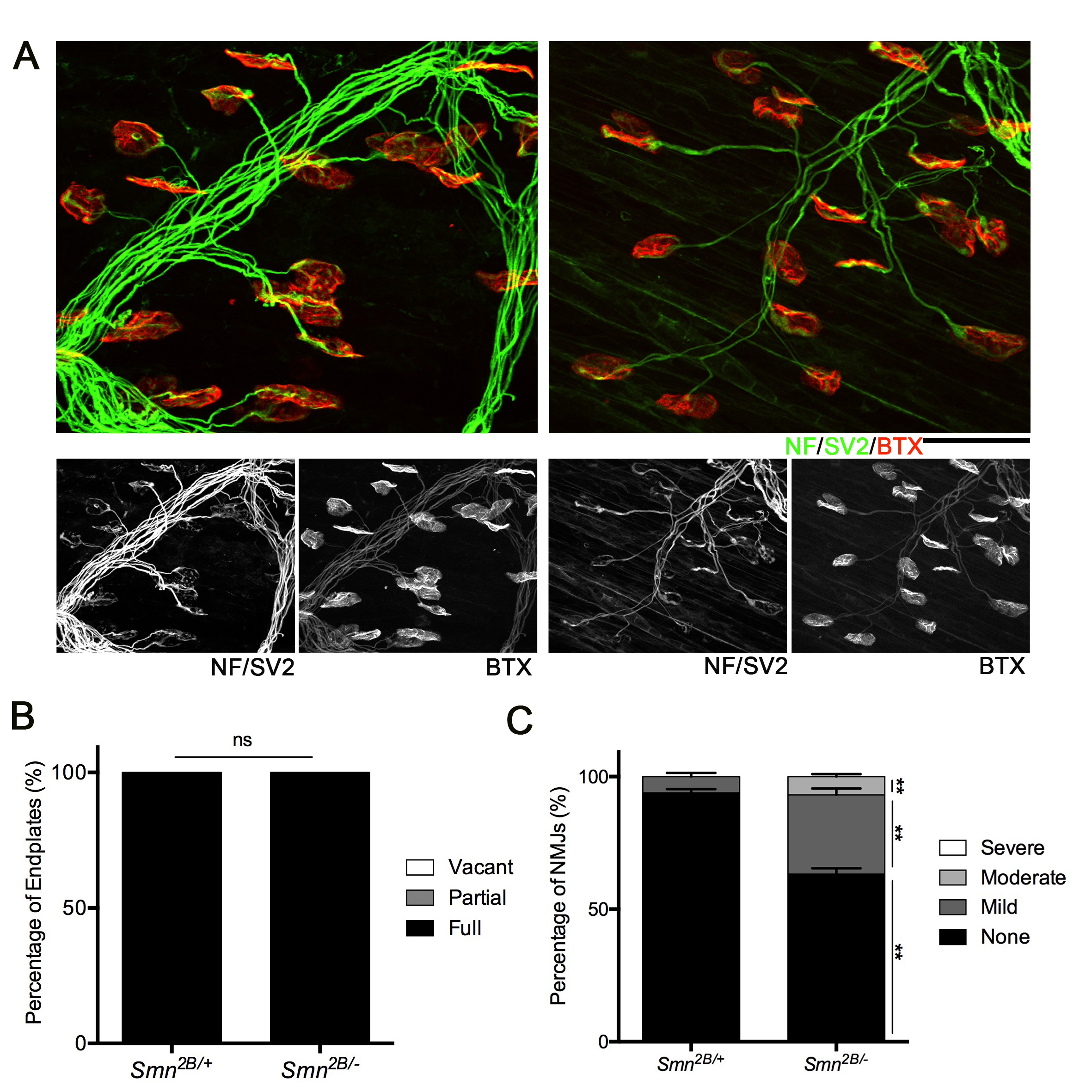

Supplement: Supplementary file 2 — Supplementary Figure 2. [file 41419_2019_1727_MOESM2_ESM.tif]
