# Supplementary material for: Reduced P53 levels ameliorate neuromuscular junction loss without affecting motor neuron pathology in a mouse model of spinal muscular atrophy
Source: Cell Death Dis. 2019 Jul 4;10(7):515. doi: 10.1038/s41419-019-1727-6 (PMC6609617; doi:10.1038/s41419-019-1727-6)
Supplement: Supplementary file 3 — Supplementary figure legends. [file 41419_2019_1727_MOESM3_ESM.docx]

**­­Supplementary Figure 1: Phenotypic analysis of the *Smn^2B/-^* mouse model of SMA shows that it becomes significantly different from control littermates around P9 to P10.** (**A**) Representative images showing a *Smn^2B/-^* mouse and a control *Smn^2B/+^* littermate at P10 and P15. Note that the yellow arrowhead highlights a short dark tail and the green arrowhead highlights small ears, both of which are distinguishing features of the *Smn^2B/-^* mouse. (**B**) The graph (Mean$\pm$SEM) shows that the weights of *Smn^2B/-^* mice are significantly different from their *Smn^2B/+^* littermate controls at P10 with an increase in the level of significance thereafter (by Unpaired T-test; *p<0.05, **p<0.01, ***p<0.005, ****p<0.001; n=4 mice per genotype). (**C**) The graph (Mean ± SEM) shows the performance in time to right for *Smn^2B/-^* and *Smn^2B/+^* mice between P8 and P12. There was a significant difference in the time to right of *Smn^2B/-^* mice compared to *Smn^2B/+^* mice at P9 (by Unpaired T-test; *p<0.05; n=4 mice per genotype). Although the other time points were not statistically significant, we note that there is a clear separation in the time it took for *Smn^2B/-^* and *Smn^2B/+^* mice to right themselves between P8 and P12.

**Supplementary Figure 2: Pre-synaptic swelling with no denervation in the tibialis anterior muscle at P10.** (**A**) Representative confocal images showing maximum intensity projection of confocal z-stack of NMJs in the tibialis anterior of *Smn^2B/-^* and *Smn^2B/+^* mice at P10. (**B**) Bar chart (Mean$\pm$SEM) compares the percentage of full, partial and vacant endplates in *Smn^2B/-^* mice compared to *Smn^2B/+^* controls. There was no significant difference (by Mann Whitney-U test, ns, non-significant, n=4 mice per genotype) (**C**) Bar chart (Mean$\pm$SEM) compares the stages of pre-synaptic swelling in *Smn^2B/-^* and *Smn^2B/+^* mice. There was a significant increase in the percentage of NMJs with mild or moderate swelling, with a decrease in the percentage of normal NMJs (by Mann Whitney-U test, **p<0.01; n=4 mice per genotype).
